# Impact of Controlling Nutritional Status Score on Mortality in Elderly Patients with Idiopathic Pulmonary Fibrosis

**DOI:** 10.3390/jcm13102825

**Published:** 2024-05-10

**Authors:** Yuji Iwanami, Kento Ebihara, Keiko Nakao, Ryuki Kubo, Midori Miyagi, Yasuhiko Nakamura, Susumu Sakamoto, Kazuma Kishi, Ikuko Okuni, Satoru Ebihara

**Affiliations:** 1 Department of Rehabilitation Medicine, Toho University Omori Medical Center, 6-11-1 Omori-nishi, Ota-ku, Tokyo 143-8541, Japan; yuuji.iwanami@med.toho-u.ac.jp (Y.I.); kento.ebihara@med.toho-u.ac.jp (K.E.); keiko.yamasaki@med.toho-u.ac.jp (K.N.); ryuki.kubo@med.toho-u.ac.jp (R.K.); pon1990@med.toho-u.ac.jp (I.O.); 2 Department of Rehabilitation Medicine, Tohoku University Graduate School of Medicine, 1-1 Seiryo-machi, Aoba-ku, Sendai 980-8574, Japan; midori.miyagi.b5@tohoku.ac.jp; 3 Department of Respiratory Medicine, Toho University School of Medicine, 6-11-1 Omori-nishi, Ota-ku, Tokyo 143-8541, Japan; yasuhiko.nakamura@med.toho-u.ac.jp (Y.N.); susumu1029@med.toho-u.ac.jp (S.S.); kazuma.kishi@med.toho-u.ac.jp (K.K.)

**Keywords:** nutritional evaluation, malnutrition, idiopathic interstitial pneumonia

## Abstract

**Background**: There are only a few reports on the nutritional status and mortality of patients with idiopathic pulmonary fibrosis (IPF). As such, this study aims to investigate the relationship between controlling nutritional status (CONUT) and the mortality of elderly patients with IPF. **Methods**: A total of 170 IPF patients aged ≥65 years old who visited the rehabilitation department of our hospital between July 2014 and July 2021 (mean age: 75.7 ± 6.3 years, sex (male/female): 138/32, %FVC: 78.3 ± 18.3%) were retrospectively analyzed. The Kaplan–Meier method and log-rank test were applied. Furthermore, using a Cox proportional hazards model with multivariate analysis, we analyzed the relationship between all-cause mortality and baseline characteristics including CONUT. **Results**: Based on the CONUT score, the normal group included 101 cases, the mild group included 58 cases, the moderate group included 11 cases, and the severe group had 0 cases. There were 49 cases of all-cause mortality events, suggesting that the mortality of the moderate group was significantly poorer than that of the normal and mild groups (*p* < 0.05). Furthermore, multivariate analysis identified GAP stage (HR: 5.972, 95%CI: 2.901~12.291, *p* < 0.0001), mMRC scale (HR: 0.615, 95%CI: 0.389~0.971, *p* = 0.009), and CONUT (HR: 2.012, 95%CI: 1.192~3.395, *p* = 0.037) as factors significantly influencing mortality. **Conclusions**: Severe malnutrition was not observed in elderly patients with IPF. Moderate malnutrition was associated with a significantly higher risk of all-cause mortality, suggesting that CONUT is an important indicator for predicting mortality.

## 1. Introduction

Idiopathic pulmonary fibrosis (IPF) is the most common type of idiopathic interstitial pneumonia and its prevalence increases with age. It is a chronic, progressive, and irreversible disease with poor mortality due to advanced fibrosis. IPF is characterized by a pattern known as usual interstitial pneumonia (UIP pattern), usually observed on high-resolution computed tomography (HRCT) and via pathological findings [1,2].

Nutritional evaluation is important for patients with chronic respiratory diseases because they are often prone to malnutrition due to the aggravation of respiratory symptoms, increased energy consumption, and decreased food intake [3]. Malnutrition is an independent prognostic factor for chronic respiratory diseases such as Chronic Obstructive Pulmonary Disease (COPD) [4].

Previous studies have reported that the prevalence of malnutrition in IPF ranges from 18.5% to 55% [5,6]. Further, various studies have reported on the relationship between nutritional status and mortality for patients with IPF using BMI, lean body mass, weight loss over time, and the Geriatric Nutritional Risk Index (GNRI) [7,8,9,10]. However, BMI can be easily influenced by region, race, and lifestyle, among other factors, and studies have pointed out that measurement at a single time point is insufficient as a prognostic indicator [7,8,9]. Although previous reports have suggested that lean body mass is an independent prognostic factor for IPF, this parameter is challenging to measure since it requires specialized equipment that may not be available in some facilities. GNRI is a nutritional index calculated using serum albumin levels and body weight [11,12]. However, this index is heavily influenced by race because it uses BMI as a factor. Given that each index has its disadvantages, there is a need for a more straightforward and more versatile nutritional screening tool.

Moreover, the IPF registry in Japan shows a growing trend towards an older age of onset [13]. Generally, it is common for older individuals to experience malnutrition and a decline in physical function, which is often characterized by frailty. Frailty has also been reported in patients with IPF [14]. Frailty and malnutrition are interrelated, and the assessment of malnutrition plays a vital role in this respect as well [15].

Based on the above, controlling nutritional status (CONUT) is an objective and simple nutritional screening tool. Developed by Ignacio et al. in 2005, CONUT is a score-based nutritional screening tool that uses data from a general blood test, including serum albumin (Alb), peripheral blood total lymphocyte count (T-Lymph), and total cholesterol (T-Cho) [16]. CONUT reflects three indicators: protein metabolism, immune function, and lipid metabolism. Several studies have reported that CONUT is linked to the mortality of diseases like chronic heart failure and malignant tumors, as well as postoperative outcomes in cardiovascular and gastrointestinal surgery [17,18,19]. Other studies have reported an association with the incidence of major acute cardiovascular events (MACEs) in patients after acute myocardial infarction [20]. The CONUT score has been extensively studied in a variety of pathologies, including in post-surgical patients and those with malignancies. However, to the best of our knowledge, there are no reports on the nutritional evaluation of IPF patients using CONUT. Therefore, this study aims to clarify the relationship between CONUT and mortality in elderly patients with IPF.

## 2. Materials and Methods

### 2.1. Subjects

A retrospective study was conducted by extracting 170 patients with IPF from the 390 participants who participated in the Toho Rehabilitation for Interstitial Pneumonia (TRIP) study conducted from July 2014 to July 2017. The TRIP study examined the long-term effects of respiratory rehabilitation on patients with interstitial lung disease (ILD) [21,22,23]. We excluded patients who met the following exclusion criteria: (1) patients in an unstable condition; (2) patients with pneumomediastinum/pneumothorax; (3) ILD types other than IPF; (4) patients with dementia; (5) patients with malignant tumors; and (6) patients younger than 65 years old.

Based on the guidelines of the American Thoracic Society (ATS) and European Respiratory Society (ERS), IPF was diagnosed via a multidisciplinary discussion (MDD) in Toho University Omori Medical Center [24].

### 2.2. Measurements

#### 2.2.1. Measurements of Patient Background

Using treatment records, we obtained the age, sex, height, weight, Body Mass Index (BMI), history of long-term oxygen therapy (LOT), history of anti-fibrotic drug use, history of steroid use, history of acute exacerbation, and smoking history of the included patients.

#### 2.2.2. Controlling Nutritional Status (CONUT)

Controlling nutritional status (CONUT) is a nutritional scoring tool that uses data from a general blood test, including total albumin (Alb) score, total lymphocyte count (T-Lymph), and total cholesterol (T-Cho) [16]. The total score is classified into four levels: normal (CONUT: 0–1 points), mild malnutrition (CONUT: 2–4 points), moderate malnutrition (CONUT: 5–8 points), and severe malnutrition (CONUT: 9–12 points). Higher scores indicate poorer nutritional status.

#### 2.2.3. Pulmonary Function Test

The pulmonary function test, which includes forced vital capacity (FVC), forced expiratory volume in one second (FEV_1_), FEV_1_/FVC, and diffusing capacity of the lung for carbon monoxide (DLco), was performed according to guidelines [25,26]. Arterial blood gas analysis was conducted by collecting arterial blood while at rest and measuring it using a spectrophotometer (ABL800 Flex; Radiometer Medical, Copenhagen, Denmark).

#### 2.2.4. Severity

The multidimensional index and staging system (Gender–Age–Physiology Index Stage: GAP stage) was used to evaluate the severity of IPF [27]. Sex, age, and lung function (%FVC, %DLco) are each scored and staged according to the score. The higher the score, the higher the stage, which indicates increased severity.

#### 2.2.5. Dyspnea

We used the Modified Medical Research Council Dyspnea Scale (mMRC scale) to evaluate subjective dyspnea based on the degree of shortness of breath during daily life. The scale ranges from 0 to 4, with higher values indicating more pronounced shortness of breath and more significant limitations in everyday life [28].

#### 2.2.6. Physical Function

Quadriceps force (QF) was measured using a hand-held dynamometer (a Mobie: Sakai Medical Corp., Tokyo, Japan) to measure isometric knee extension muscle strength, as reported by Dowman, L. et al. Measurements were taken twice on each side, and the maximum value was taken as the QF. The value was then adjusted according to the participant’s body weight [29].

We measured hand grip strength twice on each side using a hand dynamometer while standing with the upper limbs extended down to the sides.

6MWT was performed according to the American Thoracic Society (ATS) guidelines [30]. However, due to facility-related constraints, we had to use a 60 m course and instructed the participants to try their best to walk for six minutes. During the test, the participants wore a pulse oximeter (TEIJIN PULSOX-M) to monitor their SpO2 and pulse rate. We assessed subjective dyspnea using the modified Borg Scale before and after the 6MWT. For patients undergoing long-term oxygen therapy, the oxygen flow rate during exertion was prescribed by the physician.

#### 2.2.7. Health-Related Quality of Life (HRQOL)

HRQOL was evaluated using the COPD Assessment test (CAT) [31]. The CAT is a simple self-administered questionnaire consisting of eight questions. Each question is evaluated on a scale of 0 to 5, with a total score of 40 points, and higher scores indicating worse health.

### 2.3. Statistical Analysis

We analyzed patient outcomes using medical records from July 2014 to July 2021. Survival time was calculated as the time from initial diagnosis to death.

CONUT scores were classified into four groups based on their respective scores: normal group (CONUT: 0–1 points), mild group (CONUT: 2–4 points), moderate group (CONUT: 5–8 points), and severe group CONUT: 9–12 points). Each group classified by CONUT was compared for each parameter using ANOVA (post hoc Tukey’s test) and χ^2^ tests. The Kaplan–Meier method and log-rank test were then used to analyze these groups. We selected the survival period as the dependent variable, while factors that showed a significant difference in the univariate analysis using the Cox proportional hazards model were used as explanatory variables. Moreover, multivariate analysis was performed by inputting the CONUT scores. To prevent multicollinearity, only one of the highly correlated variables (Pearson’s correlation coefficient ≥ 0.6) was entered into the multivariate model. The statistical software used was SPSS version 17 (SPSS Inc., Chicago, IL, USA).

## 3. Results

**Figure 1:**
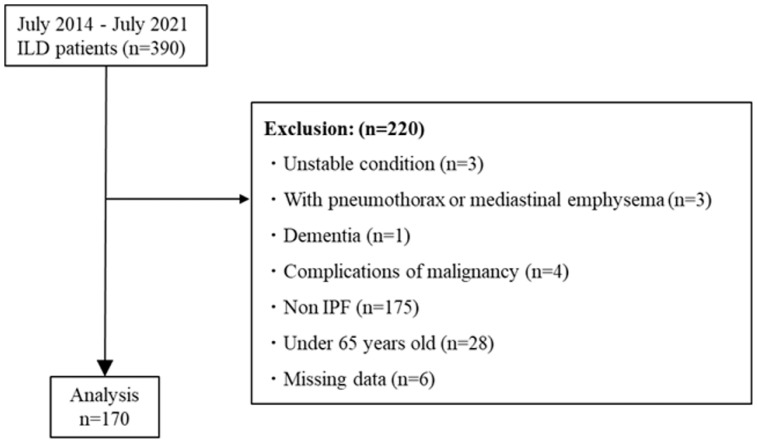
Patient flow diagram. Abbreviations: ILD, interstitial lung disease; IPF, idiopathic pulmonary fibrosis.

A total of 390 patients with ILD were recruited to the TRIP study between July 2014 and July 2021. Among them, 170 patients met the criteria for inclusion in this study (Figure 1).

The average CONUT score was 1.5 ± 1.5. Table 1 shows the characteristics of the included patients. Based on the CONUT grades (CONUT), 101/101 cases were in the normal group, 58/32 cases were in the mild group, 11/27 cases were in the moderate group, and 0/10 cases were in the severe group. Therefore, malnutrition (CONUT score ≥2) was observed in approximately 40% of the patients.

In each group classified by CONUT, age, BMI, history of steroid use, history of acute exacerbation, CONUT score, C-reactive protein (CRP), albumin (Alb), total cholesterol (T-Cho), total protein (Tp), mMRC scale, 6MWD, and hand grip were significant.

**Figure 2:**
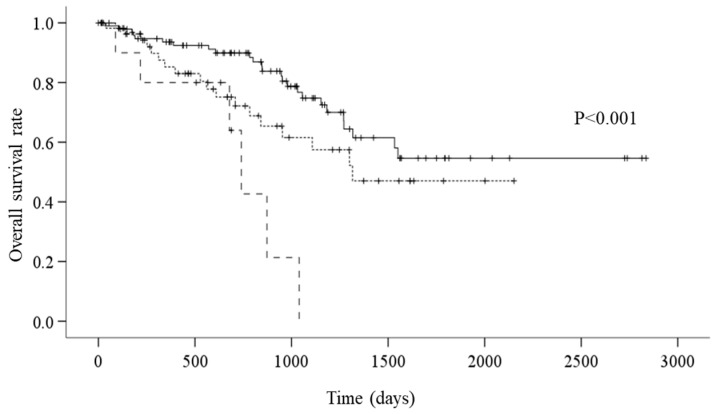
A: Kaplan–Meier survival curves according to CONUT (controlling nutritional status) (—: normal group; - - - - - - -: mild group; – – –: moderate group) (*p* < 0.001). Survival curves were compared using log-rank statistics.

The survival analysis showed that there were 49 all-cause mortality events. On average, the overall survival period was 1842.1 days. According to CONUT grades, the average survival period was 1991.3 days for the normal group, 1394.6 for the mild group, and 705.2 days for the moderate group. According to the Kaplan–Meier method and the log-rank test, the mortality for the moderate group was significantly worse than that of the normal and mild groups (Figure 2, *p* < 0.001).

In the multivariate analysis, which utilized the CONUT score, it was found that the GAP stage (HR: 5.972, 95% CI: 2.901–12.291, *p* < 0.0001), the mMRC scale (HR: 0.615, 95%CI: 0.389–0.971, *p* = 0.009), and the CONUT score itself (HR: 2.012, 95%CI: 1.192–3.395, *p* = 0.037) were associated with all-cause mortality (Table 2 and Table 3).

## 4. Discussion

This study aimed to evaluate the nutritional status of elderly patients with IPF using the CONUT score and to explore its association with mortality.

Initially, this study found that malnutrition (CONUT score ≥2) was present in approximately 40% of the participants who underwent nutritional evaluation with CONUT. Previous research has reported that between 18.5% and 55% of patients with IPF suffer from malnutrition [5,6]. The findings of this study were similar to those of a previous study, even though different criteria were used for evaluation. The study highlights the issue of malnutrition in IPF patients, which can be caused by multiple factors such as increased respiratory muscle load, the release of inflammatory mediators, hypoxemia, and lack of exercise [32]. Therefore, the early detection of malnutrition and interventions such as appropriate nutritional therapy are important [33].

This study examined the prognostic factors in elderly patients with IPF aged ≥65 years, identifying GAP stage, CONUT score, and the mMRC scale as independent prognostic factors. Both the GAP stage and mMRC scale have been recognized as prognostic factors in previous research, which confirms that the results of this study are consistent with those in elderly patients with IPF [34,35,36,37].

Higher CONUT values indicate malnutrition, which is a factor in poor mortality for IPF patients. The results of a study showed that moderately malnourished patients have significantly worse mortality compared to those who are normal or mildly malnourished. Thus, the results indicate that CONUT may be a useful prognostic factor in elderly patients with IPF.

It has been reported that the mortality of IPF patients is related to their BMI, lean body mass, and weight loss over time [7,8,9]. The BMI is known to vary based on geographical location and race. In previous studies, the average BMI was found to be 28.2 kg/m^2^. For this study, BMI was divided into three categories—<25, 25–30, and <30—and compared. However, the overall BMI in this study was found to be 23.6 kg/m^2^, which is lower than the average BMI reported in previous studies. The normal group had a BMI of 24.4 kg/m^2^, also lower than that reported in previous studies [7]. A study by Nakatsuka et al. in Japan highlighted a correlation between annual weight loss and mortality, yet no significant differences were found in baseline BMI [9]. This suggests that a single-point BMI measurement may not be adequate for predicting mortality. In this study, BMI was not identified as a prognostic factor.

There were very few IPF patients with low BMI. The average BMI was 23.6 kg/m^2^, possibly because even the moderate malnutrition group in CONUT had a normal range of BMI. In addition, patients with moderate malnutrition had low albumin levels, so it is possible that their BMI remained the same due to the effects of edema. It has been reported that around 25% of patients diagnosed with COPD, who have a normal BMI, experience a decrease in their lean body mass. Experts suggest that assessing body composition, in addition to BMI, is crucial for evaluating mortality, as lean body mass is considered to be more strongly linked to mortality than BMI alone [38]. Thus, based solely on BMI, nutritional evaluation may overlook undernourished patients with poor mortality.

In addition, the univariate analysis of GNRI, which is a simple index that can be calculated from serum albumin levels and body weight, showed a significant difference. However, this significance was not found in the multivariate analysis, and a similar nutritional index, COUNT, was selected. This may reflect the different characteristics of GNRI and CONUT. CONUT, which was used in this study, is a highly versatile evaluation tool for assessing nutritional status as it can evaluate it based on general blood sampling results such as Alb, total lymphocyte count, and T-Cho, and is said to be useful for predicting the mortality of nutritional indicators. In particular, the total lymphocyte count reflects immune capacity and will be low if the person is undernourished. A decrease in total lymphocyte count is the simplest indicator of decreased cellular immunity, and at the same time, it is thought to increase the risk of developing an infectious disease [39]. Approximately 40% of IPF-related deaths in Japan are due to acute exacerbation, and although the trigger for acute exacerbation is not clear, infectious diseases are thought to be involved [40]. From this point of view, CONUT, which is a nutritional index that includes immune function, is considered to be useful as an index for predicting the mortality of IPF.

This study, along with previous research, has made it clear that malnutrition is a predictor of poor mortality, and nutritional therapy holds a particularly crucial role for elderly patients with IPF. However, it is unclear whether nutritional therapy improves mortality. Nevertheless, diverse nutritional therapies have immense potential to reduce the morbidity and mortality associated with malnutrition and improve patient outcomes [41].

Previous studies have shown that the consumption of oily fish, yogurt, dried fruit, and fruit is associated with a lower incidence of IPF, whereas the intake of alcoholic beverages and beef was associated with an elevated risk of IPF [42,43]. Additionally, higher circulating concentrations of omega-3 fatty acids were associated with slower decline in DLco and longer transplant-free survival in patients with pulmonary fibrosis [44]. As such, the role of diet and certain bioactive food components in IPF suggests that nutritional approaches should be considered as potential complementary therapies [45]. Further research in nutritional therapy is expected in the future.

In addition, pulmonary rehabilitation is said to be even more effective in improving exercise tolerance and QOL when combined with nutritional therapy, and the importance of nutritional therapy has been shown in this respect as well [3,4,46,47].

Typically, intervention strategies for malnutrition begin with screening for malnutrition, understanding dietary habits, assessing the patient’s willingness and readiness to improve, providing useful information and resources regarding optimal nutrition, and referral to a registered dietitian [41]. From this perspective, CONUT is a simple and versatile tool that can be used as a screening method for identifying nutritional problems. In the future, it will be important to investigate whether CONUT can be applied to interstitial lung disease and not only IPF.

Finally, this study has some limitations. For instance, the study was retrospective and conducted at a single institution, so some bias may exist. Additionally, the results may have been affected by a significant number of early censored cases.

## 5. Conclusions

Moderate malnutrition is associated with poor mortality in elderly patients with IPF, indicating that malnutrition may be a critical prognostic factor in this population. Furthermore, nutritional assessment using the CONUT score is deemed valuable for predicting the course of IPF and planning comprehensive management including nutritional intervention in these patients.

## Figures and Tables

**Table 1:** Patients’ baseline characteristics.

	All Patients (n = 170)	Normal Group (n = 101)	Mild Group (n = 58)	Moderate Group (n = 11)	*p*-Value
Age (years)	75.7 ± 6.3	74.1 ± 5.8	78.1 ± 6.3 ^#^	77.8 ± 6.3	<0.001 ^a^
Gender, male/female	138/32	81/20	43/15	9/2	0.643 ^b^
BMI (kg/m^2^)	23.6 ± 3.9	24.4 ± 3.4	22.5 ± 4.2 ^§^	22.3 ± 5.4	0.005 ^a^
Oxygen use, n (%)	48 (28.2)	23 (22.7)	20 (34.4)	5 (45.4)	0.112 ^b^
Smoking status (current/former/never)	3/130/37	0/80/21	2/43/13	1/7/3	0.156 ^b^
History of acute exacerbation, n (%)	8 (4.7)	2 (1.9)	4 (6.8)	2 (18.1) ^§^	0.034 ^b^
Comorbidities, n (%)					
Diabetes	31 (18.2)	16 (15.8)	15 (25.8)	0 (0)	0.078 ^b^
Hypertension	70 (41.1)	41 (40.5)	24 (41.3)	5 (45.4)	0.952 ^b^
Dyslipidemia	39 (22.9)	23 (22.7)	16 (27.5)	0 (0)	0.136 ^b^
Cardiovascular disease	22 (12.9)	8 (7.9)	12 (20.6)	2 (18.1)	0.06 ^b^
CKD	14 (8.2)	7 (6.9)	6 (10.3)	1 (9.0)	0.748 ^b^
Medications					
Predonisolone	35 (20.5)	15 (14.8)	15 (25.8)	5 (45.4) ^§^	0.028 ^b^
Pirfenidone	28 (16.4)	12 (11.8)	13 (22.4)	3 (27.2)	0.137 ^b^
Nintedanib	22 (12.9)	13 (12.8)	8 (13.7)	1 (9.0)	0.913 ^b^
Severity					
GAP stage	1.5 ± 0.8	1.5 ± 0.7	1.6 ± 0.8	2.0 ± 0.9	0.284 ^b^
I/II/III	108/33/29	68/19/14	36/11/11	4/3/4	
GAP score	3.3 ± 1.5	3.2 ± 1.4	3.5 ± 1.6	4.2 ± 1.9	0.091 ^a^
Nutrition Status					
CONUT score	1.5 ± 1.5	0.5 ± 0.5	2.5 ± 0.6 ^#^	5.7 ± 0.7 ^#‡^	<0.001 ^a^
Pulmonary function					
FVC (%pred)	78.3 ± 18.3	80.1 ± 18.6	76.0 ± 16.9	73.1 ± 22.0	0.281 ^a^
FEV_1_ (%pred)	96.5 ± 23.1	95.0 ± 22.4	99.1 ± 24.3	97.0 ± 25.0	0.590 ^a^
FEV_1_/FVC	84.7 ± 9.9	84.5 ± 10.8	84.9 ± 8.3	85.5 ± 9.5	0.949 ^a^
TLC (%pred)	77.0 ± 16.1	77.1 ± 15.5	75.8 ± 15.3	81.9 ± 25.0	0.559 ^a^
DLco (%pred)	62.9 ± 20.2	62.5 ± 19.1	64.4 ± 20.6	58.9 ± 31.1	0.750 ^a^
PaO_2_, cmH_2_O	82.5 ± 17.3	81.9 ± 14.4	84.2 ± 0.4	79.6 ± 24.6	0.609 ^a^
PaCO_2_, cmH_2_O	41.0 ± 5.7	41.1 ± 6.4	41.0 ± 4.4	39.2 ± 4.7	0.585 ^a^
Biochemical data					
CRP, mg/dL	0.6 ± 1.3	0.4 ± 1.0	0.7 ± 1.1	1.9 ± 2.8 ^§∫^	0.001 ^a^
Alb, g/dL	3.7 ± 0.5	3.9 ± 0.3	3.7 ± 0.4 *	2.7 ± 0.8 ^#^	<0.001 ^a^
T-Cho, mg/dL	187.8 ± 39.6	196.5 ± 36.7	176.1 ± 40.5 *	169.0 ± 42.3	0.002 ^a^
T-Lymph	1911.9 ± 751.1	2132.8 ± 665.5	1884.7 ± 2300.6	1519.2 ± 1186.5	0.313 ^a^
TP, g/dL	7.4 ± 0.7	7.5 ± 0.5	7.4 ± 0.8	6.5 ± 1.2 ^#∫^	<0.001 ^a^
KL-6	997.1 ± 647.9	973.2 ± 588.7	1067.2 ± 763.0	821.2 ± 462.3	0.490 ^a^
SP-D	236.0 ± 190.0	223.6 ± 162.6	261.1 ± 237.0	218.0 ± 142.6	0.490 ^a^
SP-A	82.5 ± 65.6	79.3 ± 59.9	90.2 ± 77.7	70.3 ± 42.7	0.551 ^a^
Dyspnea and functional status					
mMRC dyspnea	1.6 ± 1.1	1.4 ± 1.0	1.8 ± 1.2 ^§^	2.4 ± 1.2 ^§^	0.004 ^a^
0/1/2/3/4	(26/64/43/24/13)	(19/40/29/9/4)	(7/21/10/14/6)	(0/3/4/1/3)	
6 min walking test					
6MWD (m)	369.8 ± 119.1	394.7 ± 106.7	344.5 ± 127.1 ^§^	257.8 ± 108.5 *	0.001 ^a^
Peripheral muscle strength					
QF (Nm/kg)	1.24 ± 0.44	1.29 ± 0.42	1.14 ± 0.44	1.30 ± 0.55	0.116 ^a^
Hand grip	26.3 ± 7.6	28.3 ± 7.6	23.7 ± 7.0 *	21.1 ± 3.5 ^§^	<0.001 ^a^
Health-related quality of life					
CAT	14.5 ± 8.3	13.9 ± 8.0	15.7 ± 9.0	14.2 ± 6.2	0.531 ^a^

Data reported as mean ± SD or number (n). ^a^ *p*-values calculated using a one-way analysis of variance post hoc-Tukey’s. ^b^ *p*-values calculated using a χ^2^ test, §: *p* < 0.05 vs. normal group, #: *p* < 0.001 vs. normal group, *: *p* < 0.01 vs. normal group, ∫: *p* < 0.05 vs. mild group, ‡: *p* < 0.001 vs. mild group. Abbreviations: BMI, Body Mass Index; GAP stage, Gender–Age–Physiology Index stage; CONUT, controlling nutritional status; FVC, forced vital capacity; %pred, percent predicted; FEV_1_, forced expiratory volume in 1s; TLC, total lung capacity; DLco, lung diffusion capacity for carbon monoxide; CRP, C-reactive protein; Alb, albumin; T-Cho, total cholesterol; T-Lymph, total lymphocyte count; TP, total protein; SP, surfactant protein; mMRC, Modified Medical Research Council; 6MWD, six-minute walk distance; QF, quadriceps force; CAT, COPD Assessment test.

**Table 2:** Results of the univariate Cox proportional hazard model.

Univariate Analysis			
	Hazard Ratio	95%CI	*p*-Value
Age (years)	1.029	0.985–1.075	0.195
Sex, male/female	1.128	0.589–2.160	0.716
History of acute exacerbation	2.012	0.719–5.627	0.180
Use of AFD	1.715	0.981–2.998	0.059
GAP stage	2.465	1.777–3.420	<0.0001
BMI (kg/m^2^)	0.910	0.837–0.990	0.028
CONUT, grade	1.926	1.230–3.015	0.004
FVC (%pred)	0.954	0.937–0.972	0.0001
FEV_1_ (%pred)	0.975	0.960–0.989	<0.001
FEV_1_/FVC	1.026	0.998–1.054	0.060
TLC (%pred)	0.960	0.941–0.981	<0.001
DLco (%pred)	0.965	0.947–0.984	<0.0001
PaO_2_ (Torr)	0.988	0.696–1.008	0.232
PaCO_2_ (Torr)	0.990	0.935–1.048	0.727
CRP, mg/dL	1.044	0.738–1.475	0.809
Alb, g/dL	0.592	0.347–1.012	0.050
T-Cho, mg/dL	1.000	0.994–1.006	0.999
T-Lymph, /μL	1.000	0.999–1.000	0.350
TP, g/dL	0.861	0.541–1.370	0.520
KL-6, U/mL	1.000	0.999–1.000	0.590
SP-D, U/mL	1.001	1.000–1.003	0.030
SP-A, U/mL	0.996	0.988–1.004	0.300
mMRC dyspnea	1.415	1.117–1.792	0.004
6MWD (m)	0.994	0.992–0.997	<0.0001
QF (Nm/kg)	0.922	0.477–1.781	0.809
Hand grip	0.967	0.93–1.004	0.083
CAT score	1.047	1.010–1.086	0.010

Abbreviations: BMI, Body Mass Index; AFD, antifibrotic drugs; GAP stage, Gender–Age–Physiology Index stage; CONUT, controlling nutritional status; FVC, forced vital capacity; %pred, per cent predicted; FEV_1_, forced expiratory volume in 1 s; TLC, total lung capacity; DLco, lung diffusion capacity for carbon monoxide; CRP, C-reactive protein; Alb, albumin; T-Cho, total cholesterol; T-Lymph, total lymphocyte count; TP, total protein; SP, surfactant protein; mMRC, Modified Medical Research Council; 6MWD, six-minute walk distance; QF, quadriceps force, CAT, COPD Assessment test.

**Table 3:** Results of the multivariate Cox proportional hazard model.

Multivariate Analysis			
	Hazard Ratio	95%CI	*p*-Value
GAP stage	5.972	2.901–12.291	<0.001
CONUT	2.012	1.192–3.395	0.009
mMRC dyspnea	0.615	0.389–0.971	0.037

Includes age, sex, BMI, GAP stage, CONUT, mMRC scale, 6MWD, SP-D,: BMI, Body Mass Index; GAP stage, Gender–Age–Physiology Index stage; CONUT, controlling nutritional status; mMRC, Modified Medical Research Council.

## Data Availability

The data that support the findings of this study are available from the corresponding author, S.E., upon reasonable request.
